# Effects of MDMA on Extracellular Dopamine and Serotonin Levels in Mice Lacking Dopamine and/or Serotonin Transporters

**DOI:** 10.2174/157015911795017254

**Published:** 2011-03

**Authors:** Y Hagino, Y Takamatsu, H Yamamoto, T Iwamura, D. L Murphy, G. R Uhl, I Sora, K Ikeda

**Affiliations:** a Division of Psychobiology, Tokyo Institute of Psychiatry, Tokyo 156-8585, Japan; b Faculty of Pharmaceutical Sciences, Matsuyama University, Matsuyama 790-8578, Japan; c Laboratory of Clinical Science, National Institute of Mental Health, National Institutes of Health, Bethesda, Maryland, USA; d Molecular Neurobiology Branch, Intramural Research Program, National Institute on Drug Abuse, National Institutes of Health, Baltimore, Maryland, USA; e Department of Biological Psychiatry, Tohoku University Graduate School of Medicine, Sendai 980-8574, Japan

**Keywords:** MDMA, serotonin transporter, dopamine transporter, knockout, microdialysis.

## Abstract

3,4-Methylendioxymethamphetamine (MDMA) has both stimulatory and hallucinogenic properties which make its psychoactive effects unique and different from those of typical psychostimulant and hallucinogenic agents. The present study investigated the effects of MDMA on extracellular dopamine (DA_ex_) and serotonin (5-HT_ex_) levels in the striatum and prefrontal cortex (PFC) using *in vivo* microdialysis techniques in mice lacking DA transporters (DAT) and/or 5-HT transporters (SERT). subcutaneous injection of MDMA (3, 10 mg/kg) significantly increased striatal DA_ex_ in wild-type mice, SERT knockout mice, and DAT knockout mice, but not in DAT/SERT double-knockout mice. The MDMA-induced increase in striatal DA_ex_ in SERT knockout mice was significantly less than in wildtype mice. In the PFC, MDMA dose-dependently increased DA_ex_ levels in wildtype, DAT knockout, SERT knockout and DAT/SERT double-knockout mice to a similar extent. In contrast, MDMA markedly increased 5-HT_ex_ in wildtype and DAT knockout mice and slightly increased 5-HT_ex_ in SERT-KO and DAT/SERT double-knockout mice. The results confirm that MDMA acts at both DAT and SERT and increases DA_ex_ and 5-HT_ex_.

## INTRODUCTION

3,4-Methylendioxymethamphetamine (MDMA, street name: ecstasy) exhibits both stimulatory and hallucinogenic properties which make its psychoactive effects unique and different from those of typical hallucinogens or psychostimulants. Under *in vitro* conditions, MDMA has been shown to increase the release of dopamine (DA), serotonin (5-HT), and norepinephrine (NE) from brain slices and prevent the reuptake of DA, 5-HT, and NE into brain synaptosomes [1-4]. MDMA binds with higher affinity to the 5-HT transporter (SERT) than to the DA transporter (DAT) [5, 6] and produces a greater release of 5-HT than DA [7-9].

*In vivo* microdialysis studies have revealed that systemic injection of MDMA increases extracellular levels of DA and 5-HT in the striatum and prefrontal cortex (PFC) [7, 10-13]. MDMA induces DA release, at least in the striatum, through several mechanisms. For example, the release of DA elicited by MDMA is hypothesized to involve both transporter- [14, 15] and impulse-dependent processes [8]. Additionally, the increased 5-HT function resulting from MDMA-induced 5-HT release has been suggested to stimulate 5-HT_2_ receptors, thereby further enhancing DA release [11, 16, 17].

Monoamine transporter knockout (KO) mice provide useful *in vivo* models to analyze the effects of psychoactive drugs. In SERT-KO mice, Begels *et al.* (1998) reported a lack of locomotor-stimulating effects of MDMA [18]. MDMA self-administration is also absent in SERT-KO mice [13]. Moreover, the ability of MDMA administration to induce (γ-aminobutyric acid transporter 1 expression in the frontal cortex and midbrain was reduced in SERT-KO mice [19]. In contrast, DAT-KO mice are hyperactive [20, 21] and display perseverative locomotor patterns [22]. MDMA decreases hyperactivity and potentiates the perseverative pattern of locomotor activity in DAT-KO mice [23]. However, the mechanisms underlying these MDMA effects have not been sufficiently elucidated.

To clarify the action of MDMA on the DAT or SERT in the striatum and PFC, we investigated the effects of MDMA on extracellular levels of DA (DA_ex_) and 5-HT (5-HT_ex_) using *in vivo* microdialysis in mice lacking the DAT and/or SERT.

## METHODS

### Animals

Wildtype and DAT-KO mouse littermates from crosses of heterozygous/heterozygous DAT-KO mice on a 129/C57 mixed genetic background served as subjects. SERT-KO and DAT/SERT double-KO mouse littermates from crosses of heterozygous DAT/homozygous SERT knockout mice on a 129/C57 mixed genetic background also served as subjects. The experimental procedures and housing conditions were approved by the Institutional Animal Care and Use Committee of Tokyo Institute of Psychiatry, and all animals were cared for and treated humanely in accordance with our institutional animal experimentation guidelines. Naive adult mice were housed in an animal facility maintained at 22 ± 2°C and 55 ± 5% relative humidity under a 12 h light/dark cycle with lights on at 8:00 a.m. and off at 8:00 p.m. Food and water were available *ad libitum. *In microdialysis experiments, male and female mice from 10 to 24 weeks old were examined.

### Surgery and Microdialysis Procedure

Mice were stereotaxically implanted, under sodium pentobarbital anesthesia (50 mg/kg, intraperitoneally), with microdialysis probes in the striatum (anterior +0.6 mm, lateral +1.8 mm, ventral -4.0 mm from bregma) or PFC (anterior +2.0 mm, lateral +0.5 mm, ventral -3.0 mm from bregma), according to the atlas of Franklin and Paxinos [24]. Twenty-four hours after implantation, the dialysis experiments were performed in freely-moving animals. Evaluation of DA_ex_ and 5-HT_ex_ has been previously described [25]. Basal levels of DA_ex_ and 5-HT_ex_ were obtained from average concentrations of three consecutive samples when they were stable.

### Drugs

Drugs were dissolved in saline and administered subcutaneously (s.c.) in a volume of 10 ml/kg. MDMA (3 and 10 mg/kg) was administered after establishment of stable baseline, and the dialysate was continuously collected for 180 min.

### Statistical Analysis

DA_ex_ and 5-HT_ex_ responses to drugs were expressed as a percentage of basal levels. Areas under the curve (AUC) of DA_ex_ and 5-HT_ex_ during the 180 min period after drug administration were calculated as the effects of drugs. AUC values of all groups were analyzed using a two-way analysis of variance (ANOVA). Individual *post hoc* comparisons were performed with Fisher’s protected least significant difference (PLSD) test. In all cases, the PLSD test was applied for multiple comparisons, and values of *p* < 0.05 were considered statistically significant. Data were analyzed with Statview J5.0 software (SAS Institute Inc., Cary, NC, USA).

## RESULTS

### Baselines of DA_ex_ and 5-HT_ex _in the Striatum and PFC

The baselines of DA_ex_ and 5-HT_ex_ in the striatum and PFC are shown in Table **1**. As previously reported [25], baselines of DA_ex_ in the striatum were significantly higher in DAT-KO and DAT/SERT-double KO mice than in wildtype mice (one-way ANOVA; *F*_3,66_ = 37.708, *p* < 0.001). Base-lines of DA_ex_ in the PFC were not different between wildtype, DAT-KO, SERT-KO, and DAT/SERT double-KO mice (one-way ANOVA; *F*_3,76_ = 0.291, *p* = 0.832). Baselines of 5-HT_ex_ were significantly higher in SERT-KO and DAT/SERT double-KO mice than in wildtype mice in both the striatum (one-way ANOVA; *F*_3,66_ = 37.716, *p* < 0.001) and PFC (one-way ANOVA; *F*_3,76_ = 47.715, *p* < 0.001).

### Effects of MDMA on DA_ex_ and 5-HT_ex_ in the Striatum

**Fig. (1):**
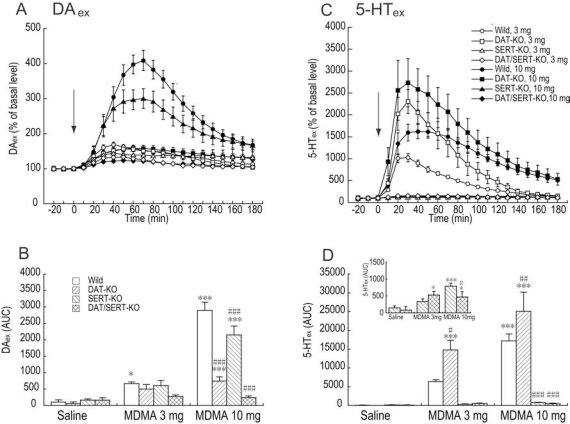
Effects of MDMA on DA_ex_ and 5-HT_ex_ in the striatum in wildtype, DAT-KO, SERT-KO, and DAT/SERT double-KO mice. (A, C) Temporal pattern of DA_ex_ and 5-HT_ex_ before and after injection with saline or MDMA (3 and 10 mg/kg, s.c.). The arrows indicate drug injection time. Each point represents the mean ± SEM of the percentage of DA_ex_ or 5-HT_ex_ baselines. (B, D) Histogram representing the mean AUC (± SEM) of DAex or 5-HT_ex_ during the 180 min period after injection with saline or MDMA (n = 4-8). ^*^p < 0.05, ^*^^*^^*^p < 0.001, compared with saline group of the same genotype; ^#^*p* < 0.05, ^#^^#^*p* < 0.01, ^#^^#^^#^*p* < 0.001, compared with corresponding wildtype data in the same drug treatment (two-way ANOVA followed by Fisher’s PLSD *post hoc* test).

MDMA (3 and 10 mg/kg) dose-dependently increased DA_ex_ in wildtype and SERT-KO mice, but not in DAT/SERT double-KO mice (Fig. **1A**, **1B**). Two-way ANOVA (drug × genotype) of the DA_ex_ AUC calculated during the 180 min posttreatment period revealed significant effects of drug (*F*_2,58_ = 94.751, *p *< 0.001) and genotype (*F*_3,58_ = 26.775,* p *< 0.001) and a significant drug × genotype interaction (*F*_6,58_ = 21.352,* p *< 0.001). *Post hoc* comparisons revealed that the effects of MDMA (10 mg/kg) on DA_ex_ in SERT-KO mice was significantly less than in wildtype mice (*p *< 0.001; Fisher’s PLSD *post hoc* test). However, DAT-KO mice exhibited significant MDMA (10 mg/kg)-induced increases in DA_ex_ levels (*p *< 0.001; Fisher’s PLSD *post hoc* test), increases that were less than in wildtype mice (*p *< 0.001; Fisher’s PLSD *post hoc* test). MDMA (3 and 10 mg/kg) dose-dependently increased 5-HT_ex_ in wildtype and DAT-KO mice (Fig. **1C**, **1D**). Two-way ANOVA (drug × genotype) of 5-HT_ex_ revealed significant effects of drug (*F*_2,58_ = 23.578, *p *< 0.001) and genotype (*F*_3,58_ = 21.589,* p *< 0.001) and a significant drug × genotype interaction (*F*_6,58_ = 7.769,* p *< 0.001). The effects of MDMA (3 and 10 mg/kg) on 5-HT_ex_ in DAT-KO mice was significantly higher than in wildtype mice (*p *< 0.05 and *p *< 0.01, respectively; Fisher’s PLSD *post hoc* test). When the effects of MDMA were analyzed only in SERT-KO and DAT/SERT double-KO mice, two-way ANOVA (drug × genotype) of 5-HT_ex_ revealed a significant effect of drug (*F*_2,25_ = 11.858, *p *< 0.001) but no effect of genotype (*F*_1,25_ = 0.492,* p *= 0.489) and no drug × genotype interaction (*F*_2,25_ = 2.773,* p *= 0.082). The effects of MDMA (10 mg/kg) on 5-HT_ex_ in DAT/SERT double-KO mice was significantly less than in SERT-KO mice (*p *< 0.05; Fisher’s PLSD *post hoc* test).

### Effects of MDMA on DA_ex_ and 5-HT_ex_ in the PFC

**Fig. (2):**
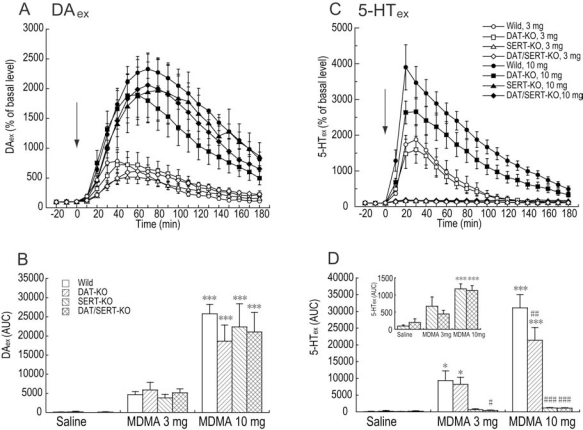
Effects of MDMA on DA_ex_ and 5-HT_ex_ in the PFC in wildtype, DAT-KO, SERT-KO, and DAT/SERT double-KO mice. (A, C) Temporal pattern of DAex or 5-HT_ex_ before and after injection with saline or MDMA (3 and 10 mg/kg, s.c.). The arrows indicate drug injection time. Each point represents the mean ± SEM of the percentage of DA_ex_ or 5-HT_ex_ baselines. (B, D) Histogram representing the mean AUC (± SEM) of DA_ex_ or 5-HT_ex_ during the 180 min period after injection with saline or MDMA (n = 4-10). ^*^p < 0.05, ^*^^*^^*^p < 0.001, compared with saline group of the same genotype; ^#^p <0.05, ^#^^#^p < 0.01, ^#^^#^^#^p < 0.001, compared with corresponding wildtype data in the same drug treatment (two-way ANOVA followed by Fisher’s PLSD *post hoc* test).

MDMA (3 and 10 mg/kg) dose-dependently increased DA_ex_ in wildtype, DAT-KO, SERT-KO, and DAT/SERT double-KO mice (Fig. **2A**, **2B**). Two-way ANOVA (drug × genotype) of DA_ex_ revealed a significant effect of drug (*F*_2,68_ = 53.368, *p *< 0.001) but no effect of genotype (*F*_3,68_ = 0.203,* p *= 0.894) and no drug × genotype interaction (*F*_6,68_ = 0.408,* p *= 0.871). MDMA (3 and 10 mg/kg) dose-dependently increased 5-HT_ex_ in wildtype and DAT-KO mice (Fig. **2C**, **2D**). Two-way ANOVA (drug × genotype) of 5-HT_ex_ revealed significant effects of drug (*F*_2,68_ = 32.357, *p *< 0.001) and genotype (*F*_3,68_ = 19.078,* p *< 0.001) and a significant drug × genotype interaction (*F*_6,68_ = 10.596,* p *< 0.001). The effect of MDMA (10 mg/kg) on 5-HT_ex_ in DAT-KO mice was significantly less than in wildtype mice (*p *< 0.01; Fisher’s PLSD *post hoc* test). When the effects of MDMA were analyzed only in SERT-KO and DAT/SERT double-KO mice, two-way ANOVA (drug ( genotype) of 5-HT_ex_ revealed a significant effect of drug (*F*_2,29_ = 28.906, *p *< 0.001) but no significant effect of genotype (*F*_1,29_ = 0.236,* p *= 0.631) and no drug × genotype interaction (*F*_2,29_ = 0.609,* p *=0.551).

## DISCUSSION

MDMA increased DA_ex_ and 5-HT_ex_ in the striatum and PFC, consistent with several previous microdialysis studies [7, 10-13]. In DAT/SERT double-KO mice, MDMA did not increase DA_ex_ in the striatum, and the increases in 5-HT_ex_ were minimal in the striatum and PFC. These results confirm that MDMA acts at both the DAT and SERT.

MDMA increased DA_ex_ in wildtype and SERT-KO mice, but not in DAT/SERT double-KO mice. In the absence of the DAT, MDMA-induced changes in DA_ex_ were smaller than in wildtype mice. Therefore, the DAT is likely mainly involved in the changes in DA_ex_ induced by MDMA. Although DAT-KO mice exhibited significant MDMA-induced increases in DA_ex_ levels, these increases were less than in wildtype mice. The increase in DA_ex_ produced by MDMA in DAT-KO mice may have two possible explanations. One possibility is that elevated 5-HT_ex_ levels produced by MDMA may influence DA release. Microdialysis studies have shown that MDMA, by increasing 5-HT_ex_, indirectly increases DA_ex_ *via* an action at 5-HT_2 _receptors [7, 8, 17]. Another possibility is that MDMA inhibits DA uptake into 5-HT axon terminals and increases DA_ex_. The SERT is able to transport DA into 5-HT cells [26, 27], and the selective SERT blocker fluoxetine increases DA_ex_ in the striatum of DAT-KO mice [25].

Microdialysis studies have shown that NET inhibitors increased DA_ex_ in the PFC [28, 29], suggesting that NET can influence DA neurotransmission. Moron *et al.* (2002) reported that DA uptake in the PFC depends primarily on the NET [30]. This study showed a similar basal extracellular DA concentration in the PFC in DAT-KO and wildtype mice. DA_ex_ in the PFC is regulated by the NET. MDMA dose-dependently increased DA_ex _in wildtype, DAT-KO, SERT-KO, and DAT/SERT double-KO mice. Therefore, MDMA may act at the NET and increase DA_ex_ levels in the PFC.

MDMA slightly increased 5-HT_ex_ in the striatum and PFC in mice lacking the SERT. The selective DAT blocker GBR12909 produced a substantial increase in dialysate 5-HT in SERT-KO mice that was not found in wildtype mice [25]. When the SERT is disrupted in SERT-KO mice, 5-HT is found in DA neurons in the substantia nigra and ventral tegmental area [31]. The DAT appears to play a compensatory role in 5-HT uptake in SERT-KO mice. Therefore, MDMA may act at the DAT and increase 5-HT_ex_ levels in the striatum in SERT-KO mice. The NET also appears to be able to play a role in 5-HT uptake [32]. In the PFC, MDMA may increase 5-HT_ex_ levels by acting at the NET in SERT-KO mice.

MDMA markedly increased 5-HT_ex_ in wildtype and DAT-KO mice. MDMA binds with higher affinity to the SERT than to the DAT [5, 6]. Consistent with *in vitro* results, MDMA produced greater elevations in 5-HT than DA. Relevant studies have shown that many of the subjective effects of MDMA in human volunteers are reduced after administration of a 5-HT_2_ receptor antagonist or 5-HT reuptake inhibitors, suggesting that these effects are dependent on SERT-mediated enhancement of serotonergic transmission [33, 34].

In conclusion, the present microdialysis study using DAT- and/or SERT-KO mice demonstrated that MDMA targets monoamine transporters and stimulates predominantly serotonergic transmission.

## Figures and Tables

**Table 1:** The Baselines (fmol/10 min) of DA_ex_ and 5-HT_ex_ in the Striatum and PFC

	**Striatum**			**PFC**		
**Genotype**	**n**	**DA_ex_**	**5-HT_ex_**	**n**	**DA_ex_**	**5-HT_ex_**
Wildtype	20	43.00 ± 5.15	1.24 ± 0.17	24	1.24 ± 0.18	1.87 ± 0.20
DAT-KO	19	486.26 ± 62.00^***^	1.01 ± 0.13	21	1.16 ± 0.16	1.87 ± 0.24
SERT-KO	16	56.18 ± 7.44	13.07 ± 1.97^***^	16	1.32 ± 0.28	15.09 ± 1.73^***^
DAT/SERT-KO	15	596.18 ± 73.38^***^	15.13 ± 1.91^***^	19	1.42 ± 0.22	12.21 ± 1.43^***^

Data presented are means ± S.E.M.

*** P < 0.001 compared to the corresponding wildtype datum.
